# Innate cellular sources of interleukin-17A regulate macrophage accumulation in cigarette- smoke-induced lung inflammation in mice

**DOI:** 10.1042/CS20140703

**Published:** 2015-08-05

**Authors:** Steven Bozinovski, Huei Jiunn Seow, Sheau Pyng Jamie Chan, Desiree Anthony, Jonathan McQualter, Michelle Hansen, Brendan J. Jenkins, Gary P. Anderson, Ross Vlahos

**Affiliations:** * School of Health Sciences and Health Innovations Research Institute, RMIT University, Bundoora, VIC 3083, Australia; † Lung Health Research Centre, Department of Pharmacology & Therapeutics, The University of Melbourne, VIC 3010, Australia; ‡ Hudson Institute of Medical Research, Monash University, Clayton, VIC 3168, Australia

**Keywords:** chronic obstructive pulmonary disease (COPD), chronic lung disease, cigarette smoke, innate immunity, interleukin-17, macrophage

## Abstract

Cigarette smoke (CS) is the major cause of chronic obstructive pulmonary disease (COPD). Interleukin-17A (IL-17A) is a pivotal cytokine that regulates lung immunity and inflammation. The aim of the present study was to investigate how IL-17A regulates CS-induced lung inflammation *in vivo*. IL-17A knockout (KO) mice and neutralization of IL-17A in wild-type (WT) mice reduced macrophage and neutrophil recruitment and chemokine (C-C motif) ligand 2 (CCL2), CCL3 and matrix metalloproteinase (MMP)-12 mRNA expression in response to acute CS exposure. IL-17A expression was increased in non-obese diabetic (NOD) severe combined immunodeficiency SCID) mice with non-functional B- and T-cells over a 4-week CS exposure period, where macrophages accumulated to the same extent as in WT mice. Gene expression analysis by QPCR (quantitative real-time PCR) of isolated immune cell subsets detected increased levels of IL-17A transcript in macrophages, neutrophils and NK/NKT cells in the lungs of CS-exposed mice. In order to further explore the relative contribution of innate immune cellular sources, intracellular IL-17A staining was performed. In the present study, we demonstrate that CS exposure primes natural killer (NK), natural killer T (NKT) and γδ T-cells to produce more IL-17A protein and CS alone increased the frequency of IL17^+^ γδ T-cells in the lung, whereas IL-17A protein was not detected in macrophages and neutrophils. Our data suggest that activation of innate cellular sources of IL-17A is an essential mediator of macrophage accumulation in CS-exposed lungs. Targeting non-conventional T-cell sources of IL-17A may offer an alternative strategy to reduce pathogenic macrophages in COPD.

## Abstract (precis)

The present study has identified IL-17A as an alternative target to combat macrophage accumulation in cigarette smoke (CS)-related lung conditions and suggests that alternative innate cellular sources should be considered when developing strategies to combat excessive IL-17A signalling in chronic lung conditions.

## CLINICAL PERSPECTIVES

• Cigarette smoke (CS) is the major cause of COPD. IL-17A is a pivotal cytokine that regulates lung immunity and inflammation. The aim of the present study was to investigate how IL-17A regulates CS-induced lung inflammation *in vivo*.
• The results of the present study show that activation of innate cellular sources of IL-17A is an essential mediator of macrophage accumulation in CS-induced lung inflammation.
• These findings suggest that targeting non-T cell sources of IL-17A may be an alternative strategy to reduce pathogenic macrophages in COPD.

## INTRODUCTION

Cigarette smoke (CS) is the major cause of chronic obstructive pulmonary disease (COPD) and accounts for ∼85% of cases in industrialized countries, but other environmental pollutants, especially biomass fuel smoke, are important causes in developing countries [1]. CS causes lung inflammation where macrophages and neutrophils are prominent, leading to oxidative stress, emphysema, small airways fibrosis, mucus hypersecretion and progressive airflow limitation [1]. Both neutrophils and macrophage lineage cells are essential effectors in these processes. However, this myelo-monocytic inflammatory response to CS responds poorly to current anti-inflammatory treatments, such as glucocorticosteroids and there is intense research to identify more effective treatments for CS-induced lung damage, particularly in the early stages of the disease before the onset of intractable emphysema. Smoke exposure is also directly implicated in neutrophil variant asthma, ACOS (asthma–COPD overlap syndrome), interstitial lung disease, rheumatic lung disease and infectious exacerbations of these conditions.

Interleukin (IL)-17A is increasingly recognized as a fundamentally important regulator of cellular immunity and is conventionally considered to arise predominantly from the ‘Th17’ (T helper 17 cells)-specific subset of Th cells (CD4 cells), which is phenotypically and functionally distinct from Th-1 and Th-2 cells and from T regulatory (Treg) cells [2]. Now identified in both rodents and humans, in mice the initial polarization of naive Th cells towards the Th17 subtype depends on T-cell receptor signalling and the presence of transforming growth factor (TGF)-β and IL-6 [3,4]. Human Th17 cells also display similarities with mouse Th17 cells as IL-1β, IL-6 or IL-23 drive the differentiation of naive Th cells towards the Th17 subtype, whereas the data on the role of TGF-β are controversial [5,6].

Most current evidence supports IL-17A as having an important role as a pro-inflammatory cytokine uniquely positioned at the interface of innate and adaptive immunity [7]. IL-17A induces the release of secondary pro-inflammatory chemokines and growth factors in most epithelial and mesenchymal cells leading, in turn, to the recruitment and accumulation of neutrophils [8]. Specifically, it is known that IL-17A stimulates the production of C-X-C chemokines (such as chemokine (C-X-C motif) ligand 8, CXCL8), granulocyte-chemotactic protein-2 and growth-stimulatory cytokines such as granulocyte colony-stimulating factor (G-CSF) and granulocyte/macrophage colony-stimulating factor (GM-CSF) [9,10]. IL-17A, in particular, promotes neutrophil activity locally in inflamed tissues as judged by increased activity of myeloperoxidase, neutrophil elastase and matrix metalloproteinase (MMP)-9 after local administration of recombinant IL-17 protein [11,12].

It is now becoming apparent that cell types other than Th17 can also produce IL-17A. Studies with human cells indicate that CD8 cells and γδ T-cells can be sources of IL-17A [13]. The same is true in mice in which invariant natural killer T-cells, Paneth cells and even granulocytes can produce IL-17A [14]. In contrast with the restricted expression of IL-17A, the IL-17 receptor (IL-17R) is ubiquitously expressed; hence, this pathway can modulate many cell/tissue types. A pathogenic role for IL-17A has been postulated in disorders where monocyte lineages are implicated as important effector cells [15,16]. There are now studies exploring the effects of IL-17A on monocytes, macrophages, osteoclasts and dendritic cells (DCs), where IL-17A regulated monocyte-derived cytokines [17,18].

IL-17A has also been implicated in lung diseases including asthma. IL-17A promotes recruitment and survival of airway macrophages during allergen-induced airway inflammation [19]. IL-17A is increased in asthmatic BALF, sputum and blood [20,21] and increased immunoreactivity for IL-17A in the asthmatic airway submucosa is associated with impaired lung function [21]. In IL-17A knockout (KO) mice, the allergen-induced airway hyper-reactivity to methacholine is significantly reduced [22]. Systemic blockade of IL-17A also inhibited the allergen-induced accumulation of neutrophils in the airway [23]. Moreover, IL-17A levels correlate with neutrophil counts in the sputum of moderate to severe asthmatics [24].

There is also evidence for an emerging role for IL-17A in COPD. IL-17A^+^ cells increased in bronchial submucosa of chronic smokers and stable COPD subjects [25,26]. Of interest is that patients with COPD have an increased number of NKT cells in the blood, lungs and sputum [27–29] and that NKT cells are a source of IL-17A [30]. Mechanistic studies in experimental CS models also demonstrate that genetic ablation of the IL-17R prevented mice against the development of emphysema [31]. Studies have also demonstrated that neutrophilic inflammation induced by CS exposure is potently suppressed in mice deficient in IL-17A [32] and in response to neutralization with a blocking antibody (Ab) [33].

Given that the interplay of innate immune sources of IL-17A and macrophages is poorly understood we have investigated their potential roles. In the present study, we demonstrate that IL-17A is not only required for CS-mediated macrophage accumulation but also that this process occurs in the absence of functional B- and T-cells, implicating alternative cellular sources of IL-17A in response to CS.

## MATERIALS AND METHODS

### Animals

Specific pathogen-free WT (wild-type; C57BL/6) or IL-17A deficient (IL-17A^−/−^) mice aged 7–10 weeks and weighing ∼20 g were obtained from Hudson Institute of Medical Research. Specific pathogen-free BALB/c mice (aged 7 weeks) were obtained from the Animal Resource Centre Pty. Ltd. and NOD SCID mice (NOD.CB17-Prkdc*^scid^*, aged 7 weeks) were obtained from Monash Animal Services. The animals were housed at 20°C on a 12-h light/12-h dark cycle in sterile micro-isolators and fed on a standard sterile diet of Purina mouse chow with water allowed *ad libitum*. The experiments described in the present paper were approved by the Animal Ethics Committee of The University of Melbourne and conducted in compliance with the guidelines of the National Health and Medical Research Council of Australia on animal experimentation.

### Cigarette smoke exposure

Mice were placed in an 18 litre perspex chamber in a class II biosafety cabinet and exposed to CS generated from nine cigarettes per day for 4 days, as previously described [34]. The mean total suspended particulate mass concentration in the chamber containing CS was approximately 420 mg/m^3^. Commercially available filter-tipped cigarettes (manufactured by Philip Morris) of the following composition were used: 16 mg or less of tar, 1.2 mg or less of nicotine and 15 mg or less of CO. In some experiments, mice were exposed to CS for 2 and 4 weeks. Sham-exposed mice were placed in an 18 litre perspex chamber but did not receive CS. On the fifth day, mice were killed by an intraperitoneal (ip) injection of sodium pentobarbitone (360 mg/kg, Sigma–Aldrich) and the lungs were lavaged with PBS. Group sizes of eight to 12 mice per treatment were used.

### Bronchoalveolar lavage

Lungs from each terminally anaesthetized mouse were lavaged *in situ* with a 400 μl aliquot of PBS, followed by three 300 μl aliquots of PBS as previously described [34]. The total number of viable cells in the bronchoalveolar lavage fluid (BALF) was determined, cytospins were prepared and cells were differentiated by standard morphological criteria. Whole lungs were cleared of blood via right ventricular perfusion of the heart with 5 ml of PBS, rapidly excised *en bloc*, snap-frozen in liquid nitrogen and stored at −80°C until required.

### Quantitative real-time PCR (QPCR)

Total RNA was extracted from 15 mg of whole lung tissue pooled from five to eight mice per treatment group using RNeasy Mini Kits (Qiagen), and reverse transcription with High Capacity RNA-to-cDNA Kit (Life Technologies) and triplicate real-time PCRs with Life Technologies pre-developed Taqman assay reagents and 18S rRNA internal control were done as previously described [34].

### Intranasal administration of anti-IL-17A monoclonal antibody

Mice were anaesthetized lightly by inhalation of methoxyflurane vapour and 50 μl of PBS containing isotype control [rat anti-mouse IgG2a monoclonal Ab (mAb), 50 μg/mouse, R&D Systems] or anti-IL-17A mAb (rat anti-mouse IgG2a, 50 μg/mouse, R&D Systems) administered intranasally 1 h before the first CS exposure on days 1–4 of the experimental protocol.

### Preparation of lung single-cell suspensions, antibodies and flow cytometry

Single-cell suspension was prepared as previously described [35]. Briefly, lungs were minced with scissors and incubated with PBS containing Liberase (Roche) for 45 min at 37°C in a shaking incubator. Cells were then washed with FACS buffer (PBS containing 2% FBS) and harvested by centrifugation (400 ***g*** at 4°C for 10 min) and red blood cells lysed in red blood cell lysis buffer (1.55 M NH_4_Cl, 0.1 M KHCO_3_ and 10 mM disodium EDTA, pH 7.4) for 5 min at room temperature. Cells were filtered through a 40 μm pore-size nylon net strainer, washed and resuspended in Dulbecco's modified Eagle's medium (DMEM) containing 2.5% FBS prior to surface staining. Cell counts were carried out using ethidium bromide/Acridine Orange exclusion.

To avoid non-specific binding of Abs to FcRγ ((fragment, crystallizable) receptor gamma), FACS buffer containing anti-mouse CD16/32 mAb (Mouse BD Fc Block™; 2.4G2, BD Biosciences) was added to all primary stains. All Abs were purchased from BD Biosciences unless otherwise stated. Anti-mouse Abs FITC-conjugated CD45 (eBisocience), γδ-T cell receptor (TCR) (eBioscience), phycoerythrin (PE)-conjugated CD49b, epithelial cell adhesion molecule (EpCAM) (eBioscience), major histocompatibility complex class II (MHCII) (Mouse I-A[d]), allophycocyanin (APC)-conjugated CD3, CD14 (eBioscience), cluster of differentiation 4 (CD4) (eBioscience), CD11b, TCRβ, PE-Cy™7-conjugated CD11c (eBioscience), CD19 and Pacific Blue-conjugated CD4, F4/80 (eBioscience), APC-Cy™7 conjugated CD8, APC-eFluor 780 conjugated Ly-6G (Gr-1, eBioscience). A strict gating strategy was used to determine different immune cell populations as previously described [35]. Briefly, for all cell sorting, cells were gated to exclude doublets and non-viable cells (either by propidium iodide, PI or by DAPI exclusion). Lymphocytes were gated according to size and CD4^+^ T-cells were sorted as CD4^+^, CD11c^−^, Ly6G^−^ and CD49b^−^. NK/NKT cells were sorted as CD49b^+^, CD4^−^, CD11c^−^ and Ly6G^−^. Myeloid cells were further gated as large granular cells and macrophages (including DCs) sorted as CD11c^+^, Ly6G^−^, CD4^−^ and CD49b^−^. Neutrophils were gated and sorted as Ly6G^+^, CD4^−^, CD11c^−^ and CD49b^−^. Cells were sorted on the Aria Cell Sorter (BD Biosciences). Flowjo software (Treestar) was used to analyse data.

### Intracellular flow cytometry

In addition to the gating strategy above, CD49b and TCRβ expression was used to further differentiate natural killer (NK) and natural killer T (NKT) cells for intracellular IL-17A staining. γδ T-cells were also gated using γδ-TCR and CD3^+^ markers. A total of 1×10^7^ single cells from the lungs of mice exposed to CS or sham/air for 4 days were cultured in a 24-well plate in DMEM containing 2.5% FBS and 10 mg/ml brefeldin A (eBioscience) with/without 50 ng/ml PMA and 1 μg/ml ionomycin for 4 h at 37°C in 5% CO_2_. Cells were washed twice in ice-cold PBS and harvested by centrifugation (400 ***g*** at 4°C for 10 min) before surface staining for each of the cell subsets. The following anti-mouse primary Abs were used to surface stain cell subsets; FITC-conjugated CD45 (Biolegend), γδ-TCR (eBioscience), PE-conjugated CD45 (Biolegend), γδ-TCR (eBioscience), CD49b (BD Biosciences), APC-conjugated CD3 (BD Biosciences), CD4 (eBioscience), CD11b, TCRβ (BD Biosciences), PE-Cy™7-congugated CD11c, CD45 (eBioscience) and Pacific Blue-conjugated CD4 (BD Biosciences), F4/80 (Biolegend), APC-Cy™7 conjugated CD8, Ly-6G (Gr-1, eBioscience). Cells were stained for 30 min at 4°C and non-specific binding prevented using anti-mouse CD16/32 mAb (Mouse BD Fc Block™; 2.4G2). After Ab incubation, cells were washed twice and resuspended in FACS buffer. Intracellular staining was carried out using the Fixation and Permeabilization Kit (eBioscience) according to the manufacturer's instructions. IL-17A was stained using anti-mouse PE-Cy™-7-conjugated (eBioscience) or PE-conjugated IL-17A (BD Biosciences). Cells were analysed using the LSR Fortessa (BD Biosciences) and for all cell subsets doublets were excluded by forward/side scatter width compared with height and side scatter-width compared with height and only CD45^+^ events included in the analysis. Flowjo software (Treestar) was used to analyse data.

### Statistical analyses

As data were normally distributed, they are presented as grouped data expressed as means±S.E.M.; *n* represents the number of mice. Differences were determined by one-way ANOVA followed by Bonferroni post-hoc test for multiple comparisons, where appropriate. All statistical analyses were performed using GraphPad Prism™ for Windows (version 5.03). Probability levels less than 0.05 (*P*<0.05) were taken to indicate statistical significance.

## RESULTS

### Deletion of IL-17A inhibits CS-induced BALF cellularity and expression of monocyte/macrophage chemokines

**Figure 1:**
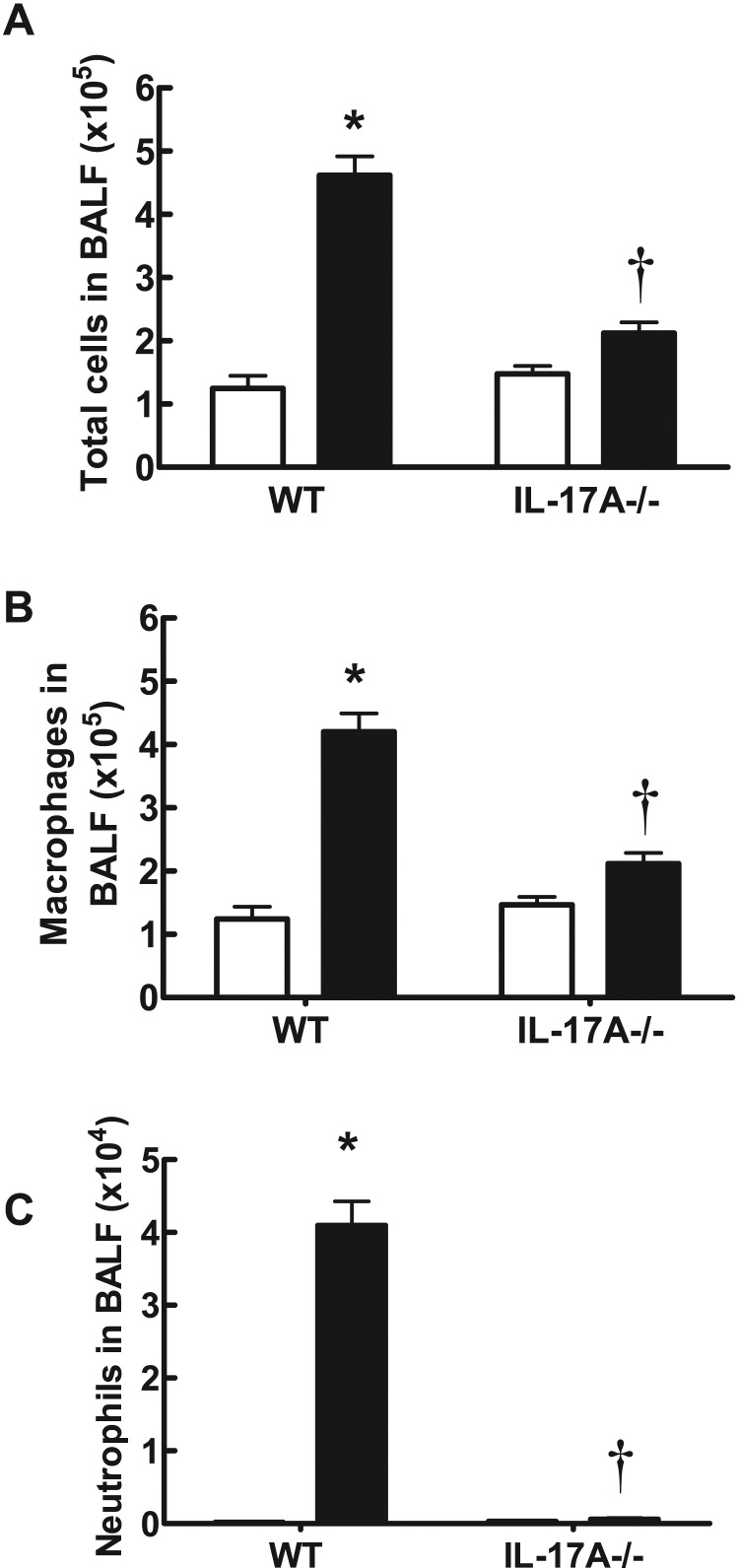
Effect of CS exposure on BALF cellularity in WT and IL-17A-/- mice IL-17A^−/−^ mice have reduced BALF total cell number (**A**), macrophages (**B**) and neutrophils (**C**) in response to 4 days of CS exposure. Data are shown as means±S.E.M. for eight to 12 mice per treatment group. White bars represent sham-exposed mice and black bars represent smoke-exposed mice. *P<0.001 compared with corresponding sham, †*P*<0.001 compared with WT smoke.

**Figure 2:**
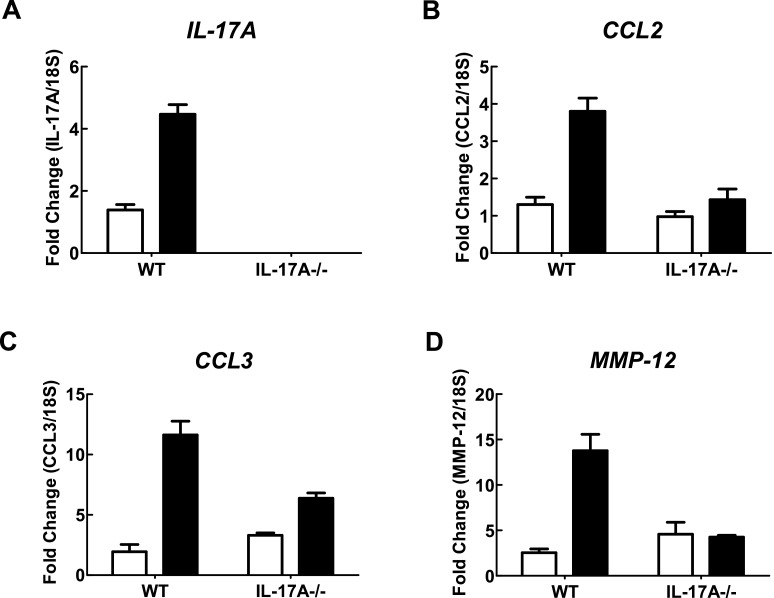
Effect of 4 days of CS exposure on whole lung gene expression in WT and IL-17A-/- mice mRNA expression for all genes was measured simultaneously under identical conditions using QPCR. Responses are shown as fold expression relative to 18S rRNA. Data are shown as means±S.E.M. of three replicates, as previously described [34]. White bars represent sham-exposed mice and black bars represent CS-exposed mice.

In WT C57BL/6 mice exposed to CS generated from nine cigarettes per day for 4 days, there was a significant increase in the total number of cells (4.62±0.29×10^5^), neutrophils (4.10±0.33×10^4^) and macrophages (4.20±0.29×10^5^) in BALF (Figures 1A–1C) compared with sham-exposed mice (1.25±0.20×10^5^, 0.02±0.010×10^4^ and 1.24±0.20×10^5^ respectively; *P*<0.05). However, IL-17A^−/−^ mice exposed to CS for 4 days had significantly fewer total cells (2.12±0.17×10^5^), neutrophils (0.060±0.01×10^4^) and macrophages (2.12±0.17×10^5^) compared with CS-exposed WT mice (Figures 1A–1C; *P*<0.05). Total cell (1.48±0.12×10^5^), neutrophil (0.03±0.02×10^4^) and macrophage (1.47±0.12×10^5^) numbers in sham-exposed IL-17A^−/−^ mice were similar to those in sham-exposed WT mice (Figures 1A–1C). WT mice treated with CS had more IL-17A, CCL2, CCL3 and MMP-12 as measured by QPCR in whole lung compared with sham-exposed WT mice (Figure 2). However, IL-17A^−/−^ mice had markedly reduced levels of IL-17A, CCL2, CCL3 and MMP-12.

### Anti-IL-17A antibody blocks CS-induced BALF phagocytic leucocyte accumulation and expression of monocyte/macrophage chemokines

**Figure 3:**
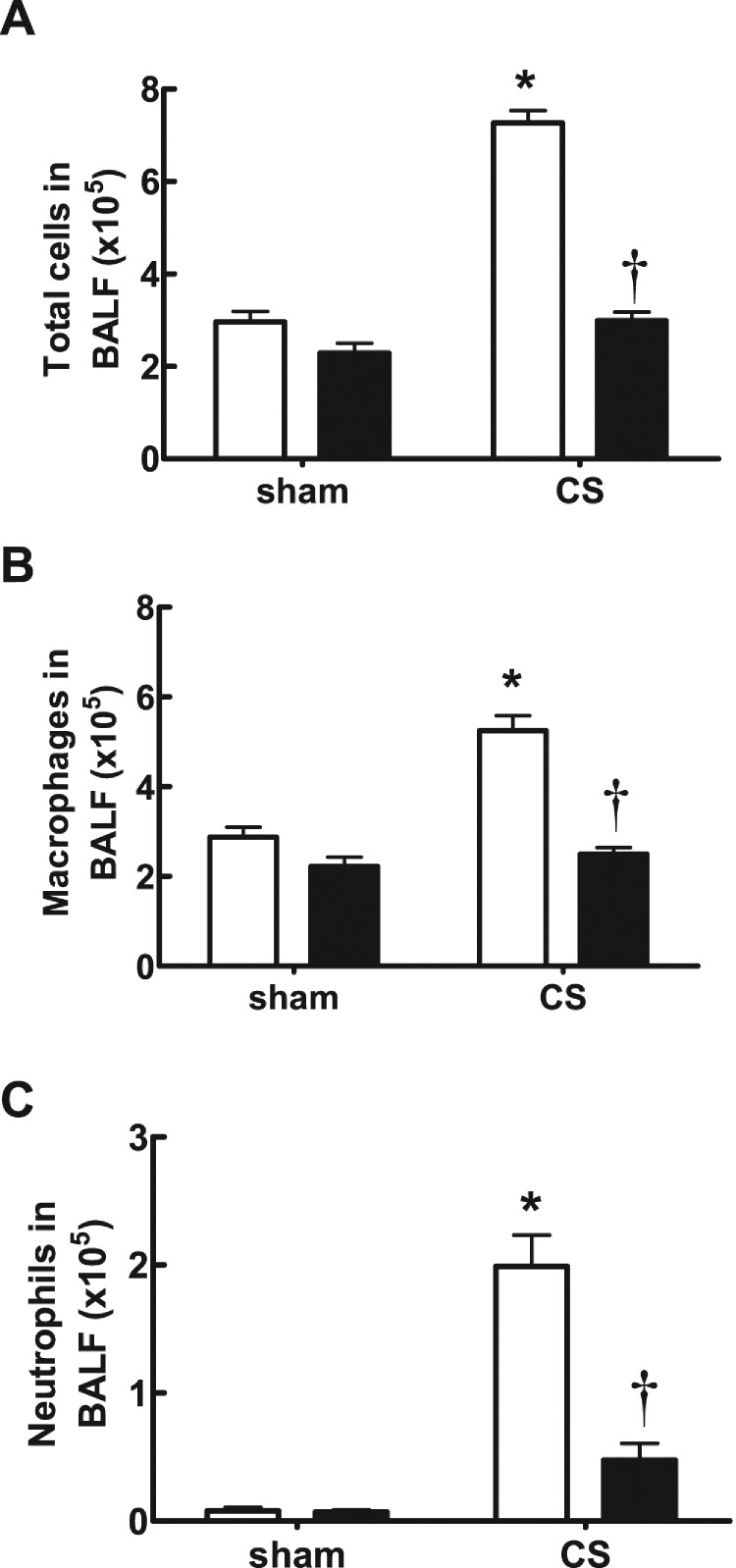
Effect of anti-IL-17A mAb on BALF cellularity in CS-exposed mice Anti-IL-17A mAb inhibits CS (4 days)-induced increases in BALF total cell number (**A**), macrophages (**B**) and neutrophils (**C**) in BALB/c mice. Data are shown as means±S.E.M. for eight mice per treatment group. White bars represent isotype control-treated mice and black bars represent anti-IL-17A mAb (50 μg/mouse)-treated mice. *P<0.001 compared with respective sham, †*P*<0.001 compared with isotype-treated CS-exposed mice.

**Figure 4:**
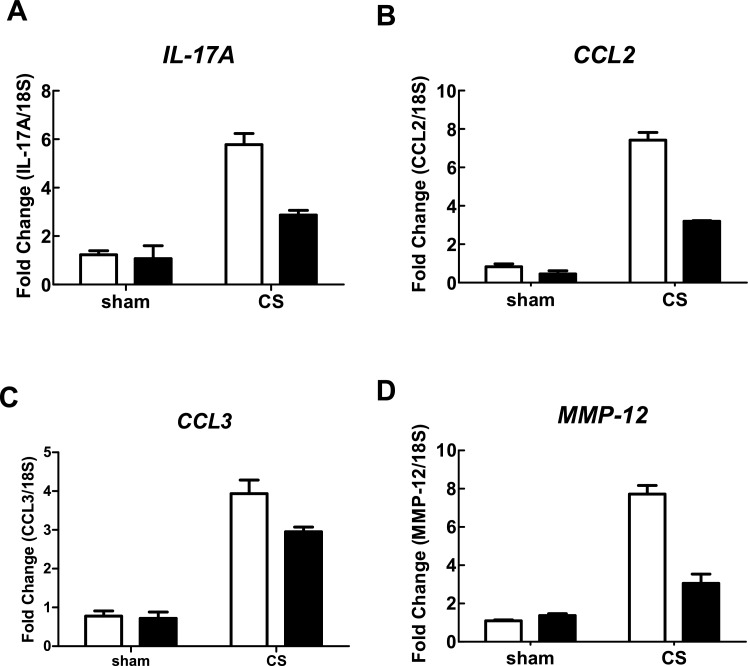
Effect of anti-IL-17A mAb on whole lung gene expression in CS-exposed mice Anti-IL-17A mAb inhibits CS (4 days)-induced increases in BALB/c whole lung IL-17A (**A**), CCL2 (**B**), CCL3 (**C**) and MMP-12 (**D**) mRNA. mRNA expression for all genes was measured simultaneously under identical conditions using QPCR. Responses are shown as fold expression relative to 18S rRNA. Data are shown as means±S.E.M. of three replicates, as previously described [34]. White bars represent isotype control-treated mice and black bars represent anti-IL-17A mAb (50 μg/mouse)-treated mice.

In CS-exposed (4 days) BALB/c mice treated with isotype control Ab, there was a significant increase in the total number of cells (7.27±0.27×10^5^), neutrophils (1.99±0.24×10^5^) and macrophages (5.250±0.33×10^5^) in BALF compared with sham-exposed isotype-treated mice (2.96±0.23×10^5^, 0.08±0.03×10^5^ and 2.88±0.22×10^5^ respectively; Figures 3A–3C; *P*<0.05). However, CS-exposed mice treated with an anti-IL-17A (50 μg/mouse) mAb had significantly reduced total cells (2.99±0.19×10^5^), neutrophils (0.48±0.13×10^5^) and macrophages (2.50±0.15×10^5^) compared with isotype-treated CS-exposed mice (Figures 3A–3C; *P*<0.05). Anti-IL-17A mAb had no effect on baseline total, neutrophil and macrophage cell numbers in sham-exposed animals (Figures 3A–3C). CS-exposed animals had markedly elevated mRNA levels of IL-17A, the chemokines CCL2 and CCL3, and the protease MMP-12 (Figures 4A–4D). Anti-IL-17 mAb (50 μg/mouse) alone had no effect on baseline mRNA levels of IL-17A, CCL2, CCL3 and MMP-12, but reduced CS-induced increases in these gene transcripts (Figures 4A–4D).

### Cigarette smoke induces IL-17A expression in the lungs of mice lacking functional T- and B-cells

**Figure 5:**
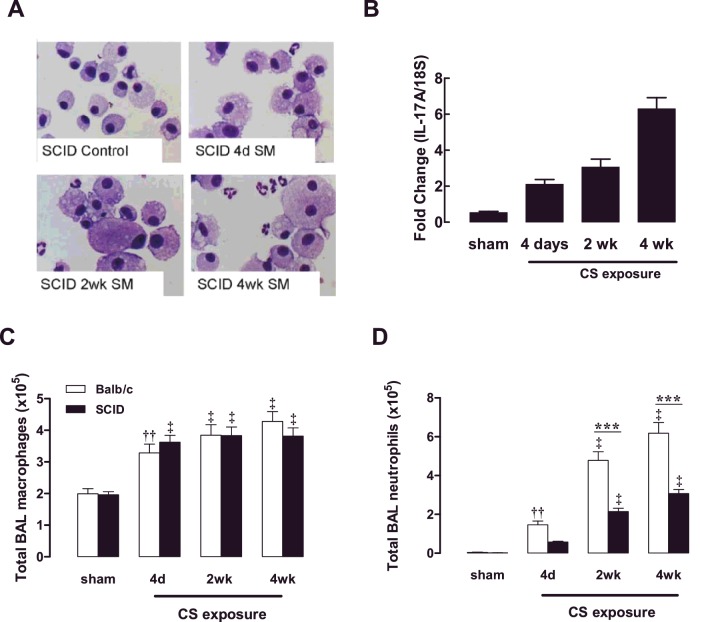
Effect of CS exposure on NOD SCID mouse BALF cellularity NOD SCID mice were exposed to CS for 4 days, 2 weeks and 4 weeks. Sham mice were not exposed to CS. DiffQuik-stained cytospins were generated from BAL cells isolated from NOD SCID (SCID) mice (**A**). IL-17A expression was measured using QPCR in NOD SCID mice at the specified time points over the 4-week CS-exposure period (**B**). The total number of BAL macrophages (**C**) and neutrophils (**D**) presented as means±S.E.M. (*n*=8–10). ††*P*<0.01, ‡*P*<0.001 compared with sham; ***P<0.001 compared with BALB/c mice.

NOD SCID mice were exposed to CS for 4 days, 2 weeks and 4 weeks. Over this 4-week CS exposure period, an increased prevalence of the foamy activated BALF macrophage phenotype was observed and neutrophilic inflammation became more prominent, as assessed by staining differential cytospots (Figure 5A). With increasing length of CS exposure, there was a progressive increase in IL-17A gene expression in the lungs of NOD SCID mice (Figure 5B). Total BALF macrophage numbers were determined in BALB/c mice and NOD SCID mice, where accumulation of macrophages in response to CS exposure was maintained in the absence of functional T- and B-cells (Figure 5C). Neutrophilic inflammation significantly progressed with increasing length of CS exposure in both BALB/c and NOD SCID mice; however, peak neutrophil numbers were reduced by approximately 2-fold in NOD SCID mice (Figure 5D).

### Alternative innate cellular sources of IL-17A in CS- exposed lungs

**Figure 6:**
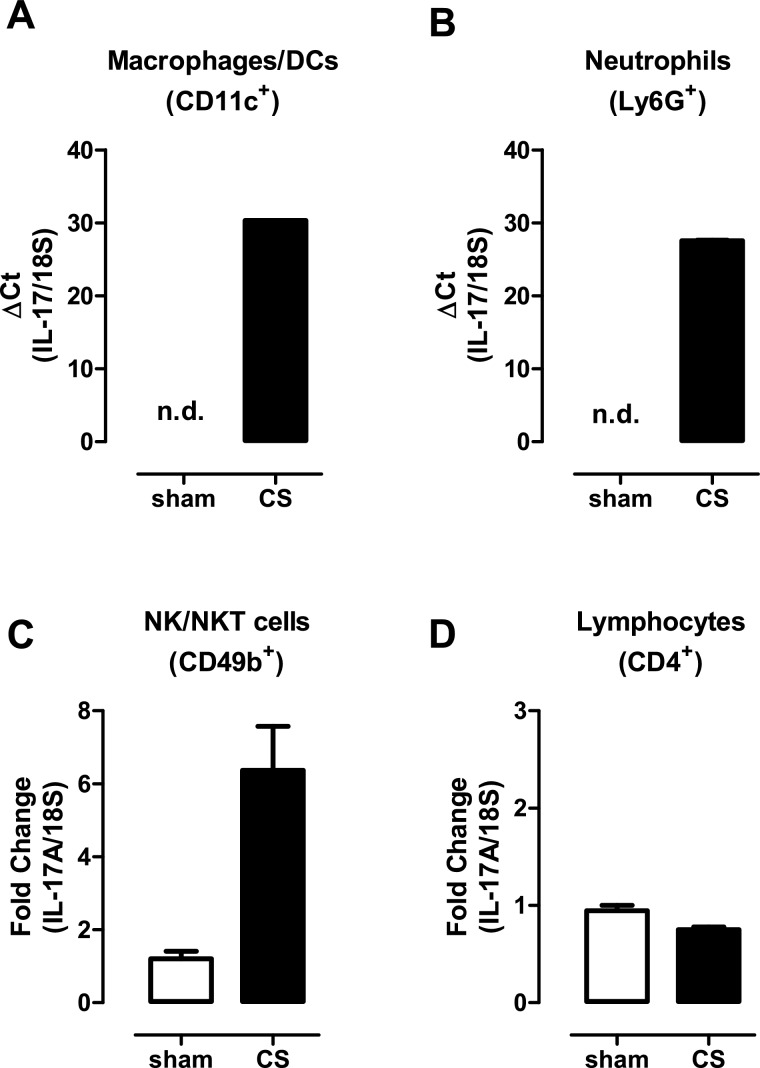
Alternative innate cellular sources of IL-17A in CS-exposed lungs Individual immune cell populations were isolated from whole lung homogenates from sham- and CS (4 days)-exposed mice as detailed in the Materials and methods section. Gene expression was determined by QPCR analysis using an IL-17A Taqman assay in sorted macrophages/DCs (**A**), neutrophils (**B**), NK/NKT cells (**C**) and lymphocytes (**D**). For analysis of macrophage/DC and neutrophil expression, Δ*C*_T_ (IL-17A–18S *C*_T_) values are presented as IL-17A expression was not detected (n.d.) in sham-exposed mice. For expression in CD4^+^ T-cells and NK cells, the ΔΔ*C*_T_ method was used as IL-17A transcript expression was detected in sham-exposed mice.

The capacity to respond to CS challenge in NOD SCID mice implicates alternative additional sources of IL-17A. To investigate these further, cell populations were sorted from the lungs of sham- and CS-exposed (4 days) BALB/c mice including macrophages, neutrophils, NK/NKT cells and CD4^+^ T-cells. Given that γδ T-cells in the lung mucosa are relatively infrequent, the number of γδ T-cells isolated from lungs was insufficient for detection of IL-17A by QPCR. The levels of IL-17A transcripts were subsequently determined in these isolated cell populations by QPCR. Using this approach, detectable IL-17A gene expression was observed in macrophages (Figure 6A) and neutrophils (Figure 6B), whereas IL-17A mRNA levels in phagocytic leucocytes isolated from control sham-exposed lung were below the limit of detection (Figures 6A–6B). Basal levels of IL-17A were detected in NK/NKT cells isolated from control lungs and this level increased 6-fold in NK/NKT cells sorted from CS-exposed lungs (Figure 6C). IL-17A transcript was also detected in CD4^+^ T-cells, but did not increase in expression in response to CS exposure (Figure 6D).

**Figure 7:**
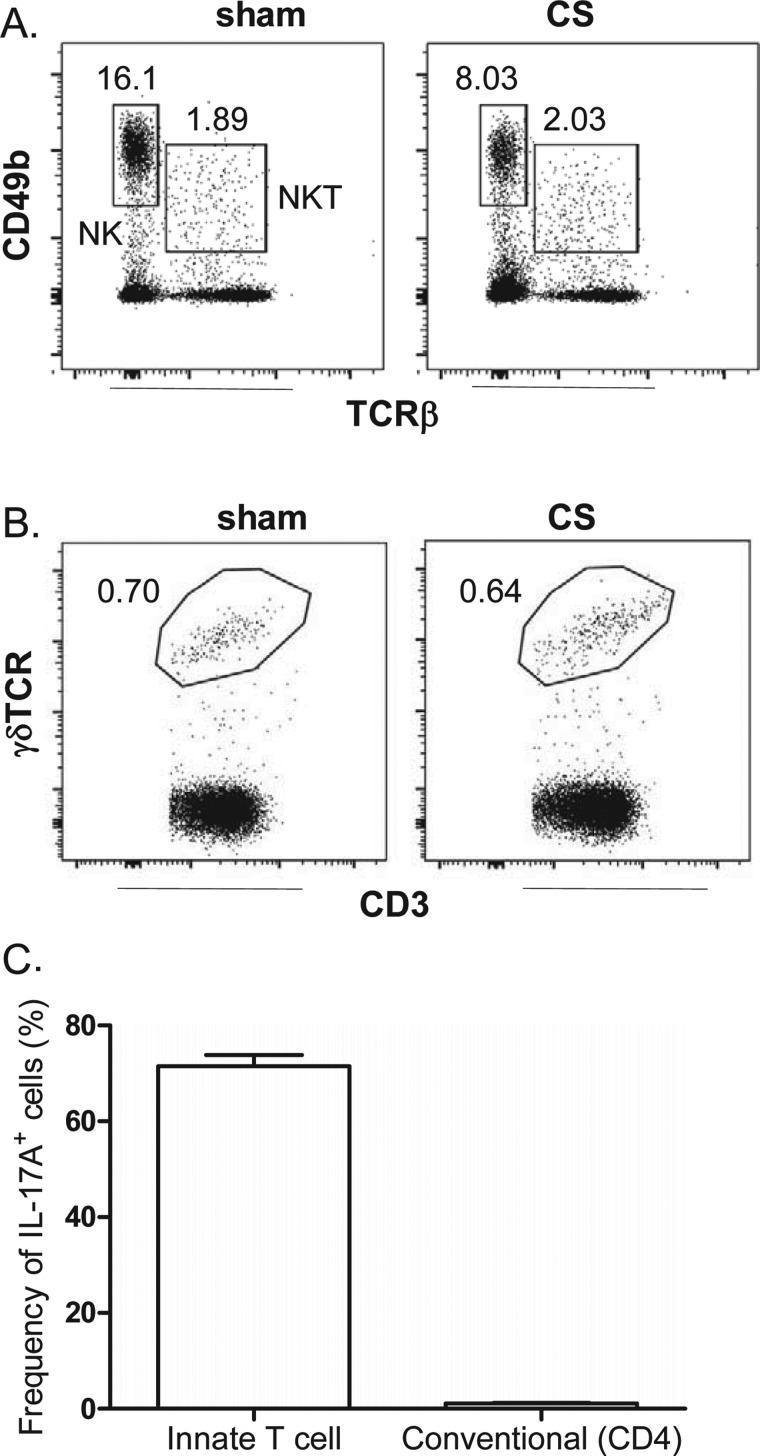
Effect of short-term CS exposure on NK, NKT and γδT-cell frequency in the lungs Lungs were harvested from mice following exposure to CS or sham for 4 days. Representative dot plots gating on (**A**) lung NK (CD49b^+^, TCRβ^−^) and NKT (CD49b^+^, TCRβ^+^) cells and (**B**) γδ T-(γδ-TCR^+^, CD3^+^) cells. Numbers represent the frequency of each cell population relative to single CD45^+^ lymphocytic events. (**C**) Single- cell suspensions from CS-exposed mice were cultured for 4 h in medium containing brefeldin with or without PMA/ionomycin and stained for intracellular IL-17A. The frequency (percentage of total IL-17A^+^ cells) of IL-17^+^ innate T-cells in innate immune cellular sources (that include NK, NKT and γδ T-cells) is compared with IL-17A^+^ conventional CD4^+^ T-cells.

To complement the QPCR experiments, intracellular IL-17A staining was performed on individually sorted immune cell populations. First, the frequency of NK, NKT and γδ T-cells was determined relative to all CD45^+^ lymphocytes in the lung of sham- and CS-exposed mice. Using CD49b and TCRβ expression to gate and differentiate NK and NKT cells, we show that acute CS exposure does not increase the frequency of these cells (Figure 7A). Indeed, a decrease in viable NK cells in lungs of CS-exposed mice (2.1±0.3×10^6^) compared with sham mice (3.4±0.14×10^6^) was observed (*P*<0.05). Likewise gating for γδ T-cells using the γδ-TCR and CD3 marker demonstrated no increase in frequency of this cell type in the lung in response to CS exposure (Figure 7B). We next investigated intracellular IL-17A staining and found that the majority of cells positive for intracellular IL-17A were innate immune cells (that included NK, NKT and γδ T-cells), whereas conventional CD4^+^ T-cells represented a minor positive population (Figure 7C).

**Figure 8:**
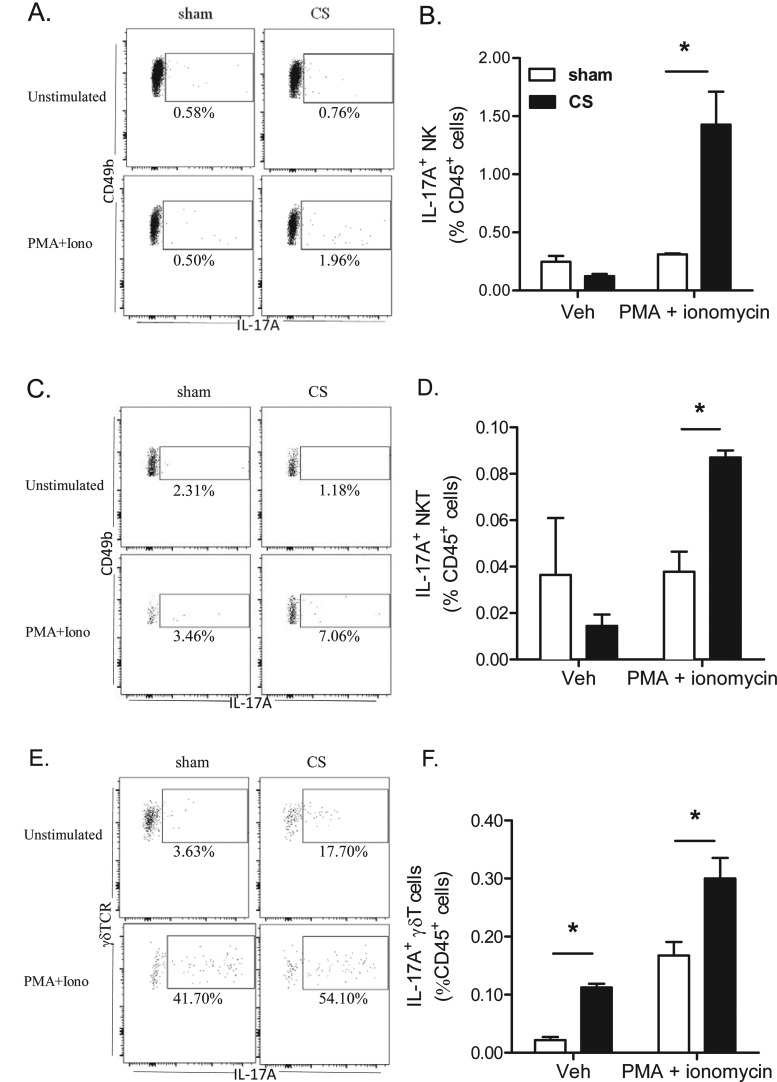
The frequency of intracellular IL-17A+ innate T-cell subsets following CS exposure Lungs were harvested from mice exposed to CS or sham for 4 days; single-cell suspensions were generated and cultured for 4 h in medium containing brefeldin with or without PMA/ionomycin prior to staining for intracellular IL-17A. Representative dot plots gating on IL-17A^+^ cells in (**A**) lung NK, (**C**) NKT cells and (**E**) γδ T-cells. Numbers represent the percentage of IL-17^+^ cells relative to each cell subset. The adjacent histograms show the frequency of IL-17A^+^ NK (**B**), NKT (**D**) and γδ T- cells (**F**) relative to all single CD45^+^ cells. Pooled data from *n*=4 mice per group. *P<0.05, as determined by two-way ANOVA.

We initially analysed several different known sources of IL-17A protein, but found that IL-17A staining was only consistently and robustly detected primarily in NK, NKT and γδ T-cells with no detectable levels in macrophages and neutrophils. In the present study, we demonstrate that 4 days of CS exposure significantly primed the release of IL-17A protein expression. Re-stimulation with PMA/ionomycin did not increase intracellular IL-17A staining in NK and NKT cells from sham-exposed mice. In contrast, the frequency of IL-17A^+^ NK and NKT cells from re-stimulated CS exposed lungs increased ∼4- and 2-fold respectively relative to sham (Figures 8A–8D). The frequency of IL-17A^+^ γδ T-cells relative to total γδ T-cells increased in lungs from 3% in sham to 17% in CS-exposed mice. Re-stimulation with PMA/ionomycin activated 50% of the γδ T-cells to produce IL-17A (Figure 8E). CS exposure also primed the release of IL-17A as the percentage of IL-17A^+^ lung γδ T-cells relative to CD45^+^ cells increased 2-fold in CS-exposed mice following re-stimulation (Figure 8F).

## DISCUSSION

The present study has shown that activation of innate cellular sources of IL-17A is an essential mediator of macrophage accumulation in CS-exposed lungs. Thus, targeting innate cellular sources of IL-17A may offer an alternative strategy to reduce pathogenic macrophages in COPD. IL-17A is a central regulator of neutrophilic inflammation during infection of the airways [36] and there is increasing evidence for its role in chronic lung diseases including asthma, COPD and cystic fibrosis. In the healthy setting, there are few IL-17A^+^ cells in the lung; however, there is evidence for their accumulation in the airway mucosa in chronic inflammatory conditions, such as COPD [25,26]. The emergence of IL-17A^+^ cells appears to be associated with disease progression as IL-17A^+^ cells are present in chronic smokers [25,26] and CS itself has been shown to be a potent inducer of Th17 cells [31]. In our model, an increase in IL-17A expression within the lung tissue was observed within 1 week of CS exposure and levels continued to increase over a 4-week exposure period. The persistence of IL-17A^+^ cells within the airway mucosa of people with chronic lung disease may not only sustain inflammation, but may also alter essential host defence to respiratory infections, leading to excessive inflammatory responses during exacerbations.

A mechanistic role for the IL-17 family has been characterized using IL-17R-deficient mice, which did not develop emphysema and did not accumulate macrophages in response to chronic smoke exposure [31]. In this study, the authors also showed that CS extract enhanced Th17 polarization of naive CD4^+^ and CD8^+^ T-cells [31]. Consistent with these findings, mRNA expression of IL-17A has been observed in CD4^+^ and CD8^+^ T-cells within the mucosa of tissue sections from COPD subjects [37,38]. In addition to the classic Th17 paradigm, the findings presented in the present study demonstrate that innate cellular sources of IL-17A can compensate for non-functional adaptive immunity. Here, NOD SCID mice, which are deficient in functional B- and T-cells, expressed increasing levels of IL-17A transcript in response to CS exposure over 4 weeks. Moreover, BALF macrophages also doubled in CS-exposed NOD SCID mice, which is equivalent to that observed in WT mice with functional B- and T-cells. Neutrophilic inflammation significantly progressed with increasing length of CS exposure in both BALB/c and NOD SCID mice; however' peak neutrophil numbers were reduced approximately 2-fold in NOD SCID mice, suggesting that conventional T-cells play an important role in the recruitment of neutrophils. In addition, NOD SCID mice continue to develop pulmonary emphysema when chronically exposed to CS as evidenced by an increase in mean linear intercept and destructive index [39], which implicates innate immune responses in pathological remodelling in COPD.

There is clinical evidence for alternative non-T cells sources of IL-17A in COPD including the identification of IL-17A^+^ macrophages and neutrophils present in the sub mucosal layer in COPD lung tissue [40]. Consistent with this finding, increased expression of IL-17A mRNA transcript was detected in both macrophages and neutrophils in our study. This finding may be particularly relevant to steroid-resistant inflammation, as neutrophils increase with disease severity despite escalating use of inhaled glucocorticoids [41]. Macrophages also develop resistance to steroids through loss of histone deacetylase 2 (HDAC2) expression in COPD [42]; hence, leucocytes may self-sustain innate inflammation through IL-17A-dependent mechanisms. Neutrophils and macrophages have also been described as an important source of IL-17A in infection-induced allergic airways disease [43]. In the present study, NK/NKT cells isolated from the lungs of CS-exposed mice also expressed IL-17A transcript.

We also evaluated IL-17A protein expression by intracellular staining using flow cytometry. Much of the focus to date has been on characterizing conventional T-cell sources of IL-17A in COPD and CS exposure models as the relative low frequency of mucosal innate cell populations has traditionally made it difficult to detect expression in these cells. Unlike mRNA transcript levels, intracellular detection of IL-17A in macrophages and neutrophils fell below the detection limit of this assay, to suggest that these cell types represent a minor contributor to IL-17A protein expression in this setting. In the present study, we demonstrate that in response to acute CS exposure, innate immune cells including NK, NKT and γδ T-cells become primed to release IL-17A and are the predominant source of IL-17A relative to conventional CD4^+^ T-cells. The role of NK cells has not been extensively characterized in COPD; however, in a chronic CS challenge model, NK cells were more primed to release inflammatory mediators including IL-12 and IL-18 [44]. The primed state of NK cells in response to CS may contribute to elevated IL-17A, as seen in our study. The NK cell group 2D (NKG2D) ligand is also elevated in CS-exposed pulmonary epithelial cells, which can sustain activation of cytotoxic T-cells and NK cells [45]. Importantly, we have recently demonstrated the persistence of NK/NKT cells and IL-17A expression in the lungs of mice chronically exposed to CS that included a 3-month cessation arm [46]. In the present study, we now formally identify NK and NKT cells as an important cellular source of IL-17A in response to CS exposure. In addition, we show that the frequency of IL-17A^+^ γδ T-cells increases even in the absence of re-stimulation. The evaluation of γδ T-cells in COPD has been difficult as this mucosal T-cell population is relatively rare; however, it is known that in response to respiratory infection such as *Streptococcus pneumoniae*, γδ T-cells can become a major source of IL-17A [47]. We believe that further assessment of γδ T-cell biology in COPD is needed in order to accurately identify dominant sources of IL-17A in this disease.

The findings of the present study demonstrate that IL-17A is required for the accumulation of airway macrophages in response to CS exposure, as assessed by use of a genetic KO mouse model and a neutralizing Ab to IL-17A. Furthermore, innate sources (macrophages, neutrophils and NK cells) of this pivotal cytokine can compensate for the loss of functional T-cells in CS-challenge models. Since macrophages are characterized as steroid-resistant immune cells in COPD, their ability to express IL-17A may sustain inflammation in a steroid-refractory manner. Macrophages in particular, can orchestrate inflammatory responses in COPD that can lead to deleterious remodelling of the airways. Airway macrophages accumulate with progression of COPD [48] and the selective depletion of macrophages in a chronic CS-exposure model using clodronate prevented pulmonary emphysema [49]. The sustained accumulation of airway macrophages in COPD is thought to be mediated by the production of chemokines that mobilize monocytes from circulation into the inflamed lungs. In the present study, both CCL2 and CCL3 were elevated in response to CS exposure, which is consistent with this concept.

Since macrophage polarization phenotypes can be highly plastic in response to external signals, the diversity of host, environmental and microbial mediators within the COPD lung microenvironment can dramatically influence the phenotype of airway macrophages. Altered macrophage phenotypes are found in COPD airways and are likely to contribute to deleterious inflammation and remodelling. MMP-12 has been identified as one of the most highly induced genes in airway macrophages of chronic smokers and represents a robust marker of an alternative activation state in both humans and mice [50]. In the present study, both genetic ablation and Ab-based neutralization of IL-17A potently suppressed MMP-12 expression in response to CS exposure, implicating IL-17A in CS-mediated macrophage polarization.

In summary, the findings of the present study identify IL-17A as an alternative target to combat macrophage accumulation in CS-related lung conditions, in addition to reducing neutrophilic inflammation. Importantly, we demonstrate that suppression of T-cell function does not effectively reduce IL-17A-mediated inflammatory responses to CS exposure. Alternative innate cellular sources should be considered when developing strategies to combat excessive IL-17A signalling in chronic lung conditions.

## Abbreviations

Ab: antibody
APC: allophycocyanin
BALF: bronchoalveolar lavage fluid
CCL: C-C (motif) chemokine ligand
COPD: chronic obstructive pulmonary disease
CS: cigarette smoke
DMEM: Dulbecco's modified Eagle's medium
IL: interleukin
IL-17R: IL-17 receptor
KO: knockout
mAb: monoclonal antibody
MMP: matrix metalloproteinase
NK: natural killer
PE: phycoerythrin
QPCR: quantitative real-time PCR
TGF: transforming growth factor
Th17: T helper 17 cell
WT: wild-type
